# Extravascular risk factors in the prognostic evaluation for spinal cord injury during thoraco-abdominal aortic aneurysm exclusion: a case report

**DOI:** 10.1186/s13019-020-01358-x

**Published:** 2020-10-17

**Authors:** Flavio Villani, Aaron Thomas Fargion, Alberto Melani, Davide Esposito, Rossella Di Domenico, Walter Dorigo, Carlo Pratesi

**Affiliations:** Department of Vascular Surgery, University of Florence, Largo Brambilla 3, 50134 Florence, Italy

**Keywords:** Collateral network, Endograft, Spinal cord ischemia, Lumbar spinal stenosis, Thoraco-abdominal aortic aneurysm, Thoracic endovascular aortic repair, Case report

## Abstract

**Background:**

The etiology of delayed-onset spinal cord injury (SCI) following endovascular repair of thoraco-abdominal aortic aneurysms (TAAA) is still unclear and may be related to multiple factors. Extravascular factors, such as lumbar spinal stenosis (LSS), may play a significant role in the selection of patient at risk of SCI. In this report we describe a case of paraplegia following thoracic endovascular aortic repair (TEVAR) in a patient suffering from severe and symptomatic LSS and undergoing staged endovascular repair of a TAAA.

**Case presentation:**

A 70-year-old man was admitted to our department with an asymptomatic type III TAAA in previous open repair for abdominal aortic aneurysm. The patient complained of buttock and thigh claudication in the absence of defects in the pelvic perfusion; a spinal magnetic resonance angiography (MRA) showed a severe narrowing of the lumbar canal.. After 24 h from first-step procedure (TEVAR) paraplegia was detected. A cerebrospinal fluid (CSF) drainage was then placed with incomplete recovery.

**Conclusions:**

Stenotic damage to the spinal cord is thought to be the result of direct compression of the neural elements and ischemic disruption of arterial and venous structures surrounding the spinal cord. This comorbidity may constitute an additional anatomic risk factor in those patients currently recognized as prognostically associated to the development of SCI.

## Background

Paraplegia secondary to spinal cord injury (SCI) remains a devastating complication following endovascular coverage of the thoracic aorta, with a reported risk of up to 20% during endovascular repair of extensive thoraco-abdominal aortic aneurysms (TAAA) [1, 2]. SCI can occur not only intraoperatively but also postoperatively, causing delayed-onset paraplegia (usually within the first 24–48 h). While the etiology of acute SCI probably lies in a state of hypoxic injury due to spinal cord hypoperfusion or collateral vessels embolism, the causes of delayed-onset SCI are still unclear and may be related to multiple factors. In order to reduce the incidence of SCI, a staged approach has been suggested [3], even though it does not represent the only available technique adopted to avoid such a complication. One of the proposed staged approaches consists of thoracic endovascular aneurysm repair (TEVAR) followed by endovascular exclusion of the visceral and infrarenal aorta with fenestrated/branched endograft. The second stage is generally considered at increased risk of SCI and requires adoption of specific SCI prevention protocols including cerebrospinal fluid (CSF) drainage. Among the factors which could potentially increase the risk of SCI, the concomitant presence of stenosis of the lumbar canal has been anecdotally reported in previous papers regarding conventional open repair of TAAA [4]; however, we are not aware of other studies describing such an association in patients undergoing endovascular treatment. In the present report we describe a case of paraplegia following TEVAR in a patient undergoing staged endovascular repair of a TAAA with a severe stenotic disease of the lumbar canal.

## Case presentation

A 70-year-old man was referred to our department for an asymptomatic thoracoabdominal aortic aneurysm. He had recently undergone percutaneous coronary procedure due to an ischemic cardiac event (myocardial infarction) and for this reason he was in treatment with a dual antiplatelet therapy (DAPT).

**Fig. 1:**
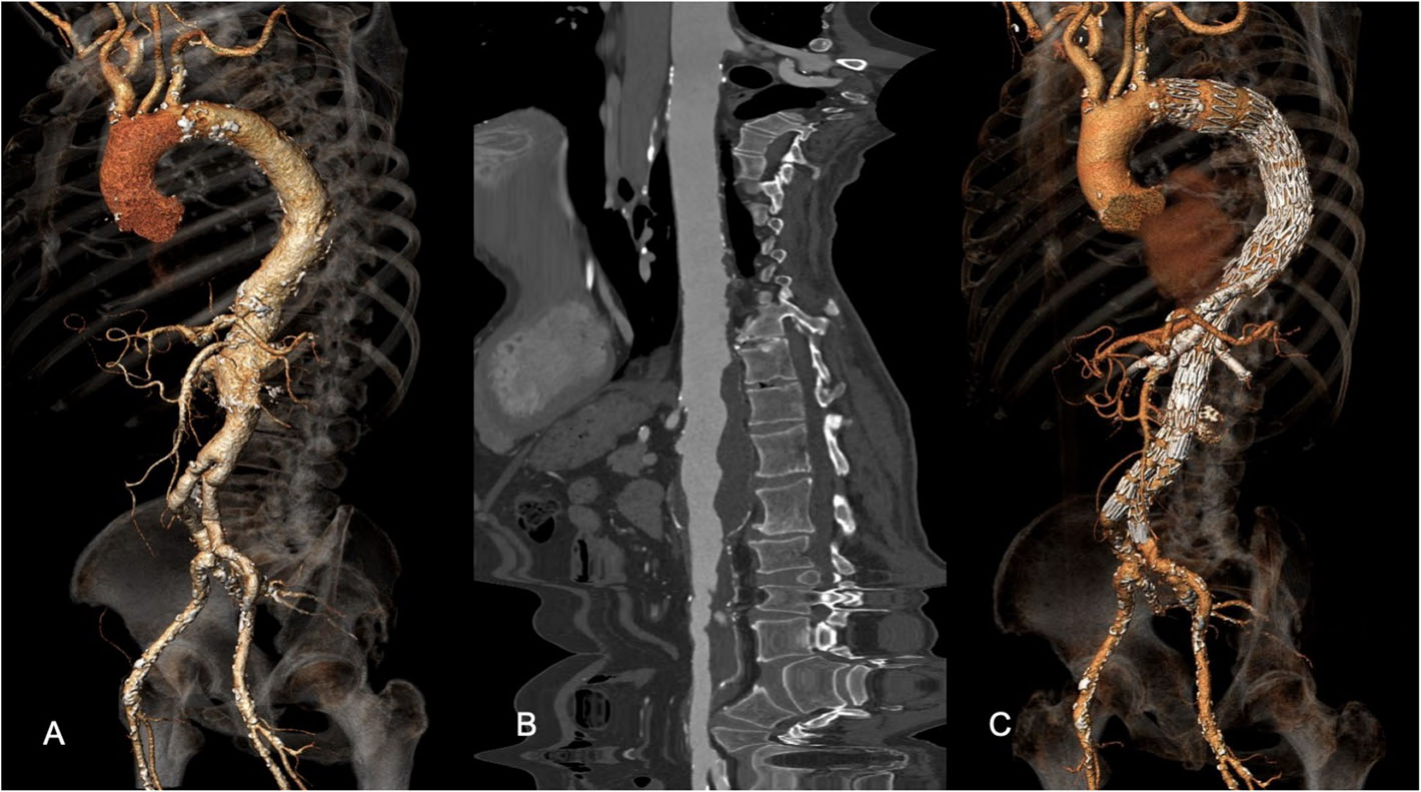
**a, b** Preoperative 3D volume rendering and vessel analysis of the CTA. **c** 3D volume rendering at 12 months showing exclusion of the TAAA and patency of the visceral vessels. Print color requested

**Fig. 2:**
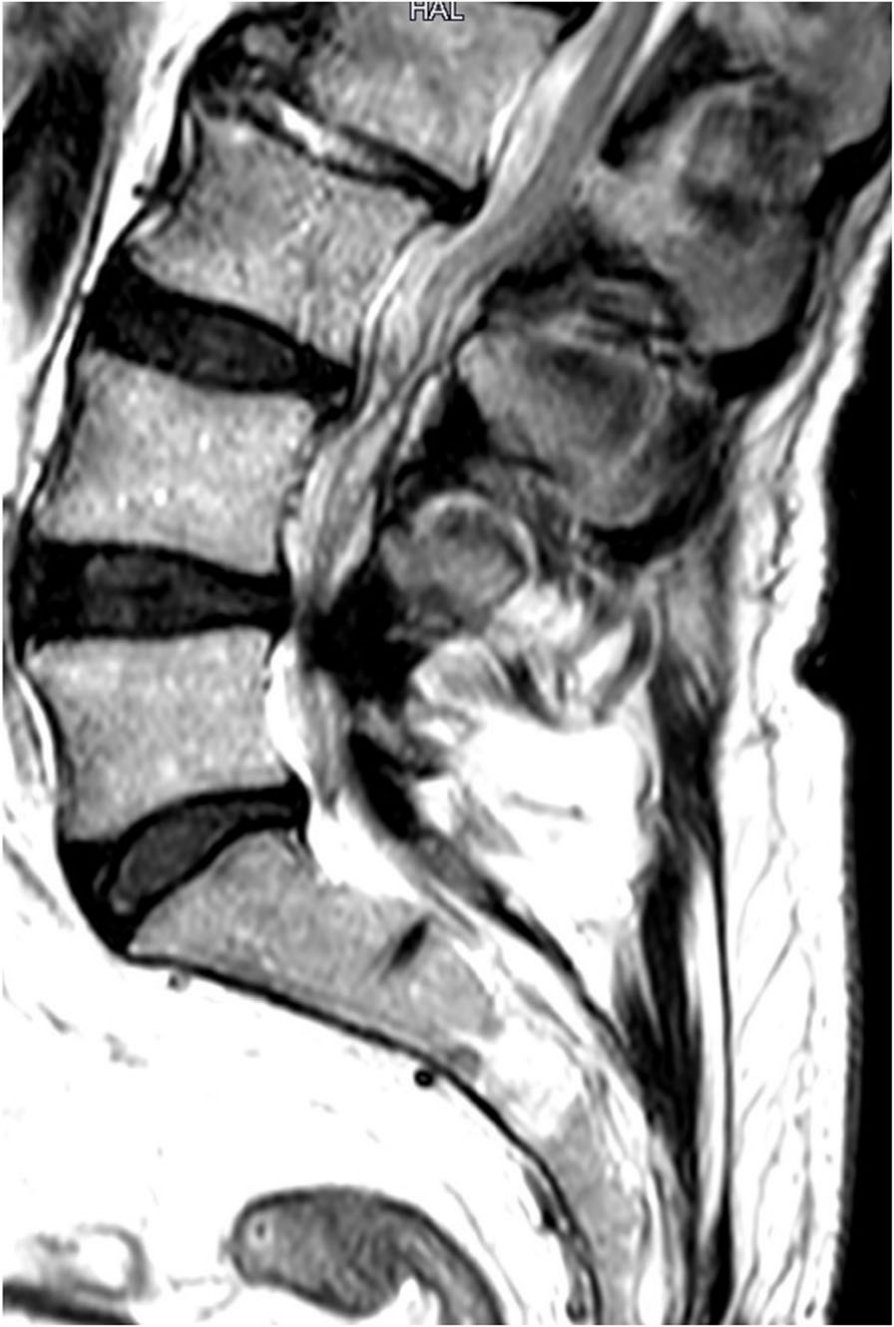
The preoperative MRA shows severe narrowing of lumbar canal stenosis

The patient, a former smoker with arterial hypertension under single medical therapy, had been previously treated for an infrarenal abdominal aortic aneurysm with open surgical repair approximately 15 years before. A computed tomography angiography (CTA) scan performed for other causes, revealed a type III TAAA with a maximum diameter of 55X65 mm (Fig. 1A and B). The patient was asymptomatic for abdominal pain, but complained of buttock and thigh claudication, even in the absence of defects in the pelvic perfusion. Symptoms were further evaluated with spinal magnetic resonance angiography (MRA) that showed a severe lumbar spinal stenosis (LSS) (Fig. 2). The patient was deemed to be at high risk for open surgery due to concomitant chronic obstructive pulmonary disease and low ejection fraction 40%) and a staged endovascular aortic repair was then planned, consisting of TEVAR followed by a second step to be performed 4 weeks later with a fenestrated custom-made endovascular repair of the visceral and infrarenal aorta**.**

Considering that subclavian and hypogastric arteries were patent and the intended length of the tract of thoracic aorta to be covered was less than 20 cm, the procedure was estimated at intermediate risk of SCI for the previous infrarenal aortic repair. Additionally, considering the need for continuing with the DAPT for the recent cardiac procedure, in agreement with the anesthesiologist, it was decided not to place a preoperative CSF drainage.

The procedure was performed with the deployment of a thoracic endoprosthesis (Cook Medical Inc., Bloomington, ZTEG-2PT-40-30-165-PF), as planned. The intervention was performed under general anesthesia, with a duration of 110 min. At the end of procedure, the patient was transferred to Intensive care Unit for postoperative monitoring, without any sign of clinical complications (Hb > 10 g/dl, mean arterial pressure (MAP) > 90, oxygen saturation > 97%). After 24 h from the procedure, incomplete paraplegia with absence of deep tendon reflex was detected. Therefore, it was decided to immediately insert a spinal catheter for CSF drainage.

CSF drainage was carried on for 7 days and the patient showed a partial recovery of the sensibility deficit (tactile dolorific hypoesthesia). The patient was transferred to the Spinal Unit, where a satisfactory recovery of the movements of the right leg was noted, whereas plegia of the left leg was still present. The patient was finally discharge in postoperative day 15th to a dedicated neurological rehabilitation center.

The second staged intervention was than delayed and, 1 week before the date of planned intervention, the patient came to the emergency department for hemoptysis and chest pain. An urgent CTA showed sudden increase of the thoracoabdominal aneurysm (78X63 mm) with contained sac rupture. The patient underwent urgent endovascular exclusion of the TAAA with the 4-branched custom made endoprosthesis: another thoracic stent-graft (Cook Medical Inc., Bloomington, ZTA-PT-36-32-161) was deployed to extend previous TEVAR. All the side branches were stented with Fluency (Bard Incorporated, Karlsruhe, Germany) and reinforced with a self-expandable stent (SMART, Cordis Endovascular, Warren, NJ). Distal abdominal stent grafting with both iliac artery extensions was performed landing distally inside the previous surgical graft.

Final digital subtraction angiography (DSA) showed complete exclusion of the TAAA and good patency of the visceral vessels. Postoperative course was uneventful. No changes in neurological status occurred. The patient was discharged in the 6th postoperative day. The CTA performed 12 months after the procedure demonstrated the continued exclusion of TAAA and the patency of visceral and renal vessels (Fig. 1C).

## Discussion and conclusions

The overall incidence of SCI (i.e., paresis or paralysis) after TEVAR ranges from to 2–10%. Other studies demonstrated a 0–10.3% SCI range, with an average of 4.5% [5]. The onset and severity of injury after TEVAR depends on the ability of the collateral network to supply the marginally vascularized area in the critical zone of the spinal cord, known as the watershed area in the grey matter of the spinal cord [5, 6]. These data show the critical nature of intercostal circle and its determining role in establishing the temporal way of onset of neurological complications. Risk factors for development of SCI after TEVAR include coverage of the left subclavian or hypogastric artery, embolization during intervention, renal failure, perioperative hypotension, prior abdominal aortic aneurysm repair, more than 3 aortic stent grafts used in the procedure, a length of total aorta to be covered of more than 20 cm [7].

The spinal cord perfusion is a terminal circulation and, as a consequence, the pathophysiology of SCI essentially follows an ischemia-infarction pattern. Aortic pathologies may alter this vascular network making it proner to hemodynamic instability. The concept of a collateral network was proposed recently [8], suggesting that multiple factors contribute to SCI, in addition to occlusion of some critical intercostal arteries, as Adamkiewitz affirmed some decades ago.

Segmental inputs from intercostal arteries are assumed to be essential for maintaining adequate flow to the anterior spinal artery, but only at certain levels do the anterior and posterior radicular-medullary arteries, which represents intercostal arteries’ last divisions, do cross the dura and reach the medulla. In fact, only a few of these segmental branches remain in adults [9].

The left subclavian artery and the internal iliac arteries also contribute to the spinal cord perfusion by delivering branches that feed the radiculo-medullary vessels.

The mechanism of SCI may involve global hypoperfusion, as with aortic cross clamping or systemic hypotension or hemodynamic instability; selective ischemia from ligation of dominant segmental vessels; or secondary insult, such as with reperfusion injury. In such patients the atherosclerotic disease and endovascular procedure may occlude many segmental arteries and promote collateral vessels enlargement, altering significantly the normal patterns of blood supply to the spinal cord, which are moreover extremely variable among individuals.

However other factors, among which extravascular components also, may play a significant role in the selection of patients at risk of developing neurological complication.

Neurologic deficits may also be related to degenerative changes of the spinal column resulting in narrowing of the spinal canal, myelopathy, or to spinal cord compression in general. Stenotic damage to the spinal cord is thought to be the result of two processes: direct compression of the neural elements and ischemic disruption of arterial and venous structures surrounding the spinal cord [10].

Several studies have shown that the radicular venous system proved to be particularly affected by the compression since the veins in the region were reduced in number or collapsed, and showed a grossly visible congestion proximal to the lesion. Congestion of the venous system, vulnerable to compression, decreases perfusion in capillaries directly feeding nerve roots, producing ischemia and consequent intra-radicular edema caused by breakdown of the blood-nerve barrier [11].

Our patient preoperative spinal MRA showed severe stenosis of lumbar canal.

It is possible that in poor circulating condition the degree of narrowing of the spinal cord could contribute to decrease of the grade of tolerance to any ischemic injury, determining a state of basal suffering which makes it more sensitive to hypoperfusion and to development of hypoxic damage. This reserve, similarly to coronary arteries, is a delicate balance between ischemic injury and capacity to meet physiological needs through activation of collateral networks and neurohormonal blood flow regulatory mechanism.

According to James et al. [4], a LSS may therefore increase the perfusion pressure required to maintain tissue perfusion to the spinal cord at the level of the stenosis, such as in this case. The fall in spinal cord perfusion pressure following loss of many contributing segmental vessels and blocked anterior spinal artery could then easily tip the balance to drop the perfusion pressure below critical level at the level of the stenosis promoting steal phenomena.

Spinal cord perfusion pressure is the difference between mean arterial blood pressure (MAP) and CSF (or central venous pressure, whichever is greater). According to general guidelines for minimizing SCI, it is advisable to increase MAP (i.e., > 90 mmHg) and drain CSF (≤ 10 mmHg) to maintain spinal cord perfusion pressure at levels above 80 mmHg [7]. However, many studies have shown that the patients with lumbar spinal stenosis have an increased epidural pressure at the level of the stenosis [12]. It may be hypothesized that patients with spinal canal disorders require an even higher blood pressure value than those proposed by standard prevention protocols, suggesting that the development of a uniform multimodal preventive treatment protocol can be elusive.

Other considerations could be done on the timing of the surgical procedure. In our case the second step was performed as an emergency by implantation of a four branched custom made endoprosthesis due to substantial rise of diameter and early sign of rupture. The onset of neurological complications led us to defer the second surgical step; an attitude of observation could be justified in order to both obtain the maximum benefits from the phenomenon of collateralization and guaranteeing a personalized treatment for the patient, an adequate preoperative planning, even if the risks deriving from postponing the treatment of a pathology that may rapidly evolve still remain a concern.

Selective reimplantation of intercostal/lumbar arteries represent an open surgical alternative performed in high-volume centers for the prevention of spinal cord ischemia. James et al. [4] documented a case in which, despite having reimplanted two pairs of intercostal arteries in a patient with post-dissecative thoracoabdominal aneurysm and concomitant stenosis of the medullary canal, irreversible medullary ischemia developed anyway. Despite to comorbidities, an open repair with selective reimplantation of the intercostal-lumbar arteries would have been rational in our case. Unfortunately, the most sensible tract of the spinal cord is the stenotic one, as theorized by some authors [10, 11] and such a surgical procedure, technically difficult and not always feasible, may not prevent SCI anyway.

In conclusion, it is likely that stenotic pathology of the medullary canal is a comorbidity that may be investigated in candidates for TAAA endovascular repair. This comorbidity may constitute an additional anatomic risk factor in those patients currently recognized as prognostically associated to development of perioperative neurological complications. The preoperative use of spinal cord MRA could be useful not only in the assessment of collateral circles but also in both the diagnosis of concomitant medullary associated diseases and the evaluation of their relative weight on the spinal injury risk. It cannot be excluded that these patients with spinal canal disorder may benefit from a personalized SCI prevention protocol.

## Data Availability

All data generated or analyzed during this study are included in this published article.

## Abbreviations

SCI: Spinal cord injury
TAAA: Thoraco-abdominal aortic aneurysms
TEVAR: Thoracic endovascular aortic repair
CSF: Cerebrospinal fluid
DAPT: Dual antiplatelet therapy;
CTA: Angiography computed tomography
MRA: Magnetic resonance angiography
LSS: Lumbar spinal stenosis
MAP: Mean arterial blood pressure
DSA: Digital subtraction angiography
